# Whole-Genome Sequencing of *Mycobacterium tilburgii* Strain MEPHI

**DOI:** 10.1128/MRA.00933-19

**Published:** 2019-10-03

**Authors:** Jamal Saad, Michel Drancourt, Margaret M. Hannan, Patrick J. Stapleton, Simon Grandjean Lapierre

**Affiliations:** a IHU Méditerranée Infection, Marseille, France; b IRD, MEPHI, IHU Méditerranée Infection, Aix-Marseille Université, Marseille, France; c Mater Misericordiae University Hospital, University College Dublin, Dublin, Ireland; d Centre de Recherche du Centre Hospitalier de l’Université de Montréal (CRCHUM), Montréal, Quebec, Canada; Loyola University Chicago

## Abstract

Mycobacterium tilburgii is a fastidious mycobacterium which has previously been reported to cause severe disseminated infections. Genome sequencing of the *M. tilburgii* MEPHI clinical isolate yielded 3.14 Mb, with 66.3% GC content, and confirmed phylogenetic placement within the Mycobacterium simiae complex.

## ANNOUNCEMENT

**FIG 1:**
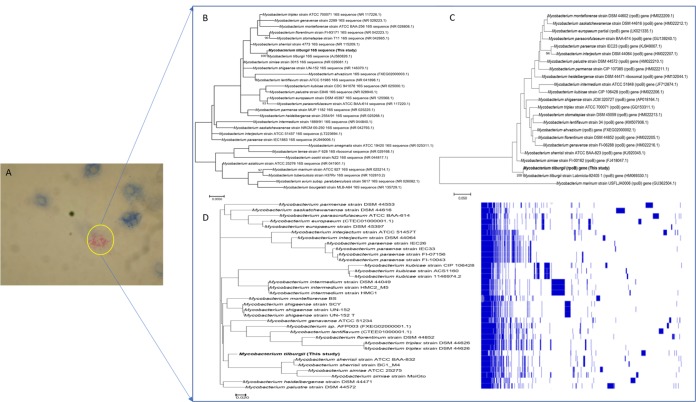
(A) Ziehl-Neelsen stain visualization of *Mycobacterium tilburgii* MEPHI strain in cell culture. (B and C) Phylogenetic tree based on the 16S rRNA gene sequence (B) and *rpoB* gene sequence (C). Sequences were aligned using Muscle v3.8.31 implemented in the MEGA7 software (13, 14). Phylogenetic inferences were obtained using the maximum likelihood method based on the Tamura and Nei model (1,000 bootstrap replicates); bootstrap values of ≥90% are given at the nodes. (D, Left) Newick tree of *Mycobacterium tilburgii* strain MEPHI and other members of the Mycobacterium simiae complex, generated by Roary from binary gene presence/absence in the accessory genome. (D, Right) Plot of Roary gene presence/absence analysis results (blue, gene presence; white, gene absence). The continuous blue block at the beginning shows the conserved genes of all members of the M. simiae complex, while regions of discontinuous blue denote accessory genome content, which varies between members.

Only 13 cases of *Mycobacterium tilburgii* infection have been reported, highlighting host immunosuppression as a risk factor for *M. tilburgii* infection (1–3). *M. tilburgii* was never previously isolated in culture; its detection relied on the direct sequencing of internal transcribed spacer 1 (ITS1), *hsp*65, *rpoB*, and 16S rRNA genes from clinical material (2). We isolated *M. tilburgii* from a bone marrow sample by using a shell vial assay, as previously described (4, 5) (Fig. 1A). This organism had been previously detected in this immunocompromised patient in a biopsy specimen taken from a duodenal plaque by direct 16S rRNA gene sequencing. DNA (0.2 μg/μl) extracted using InstaGene matrix (Bio-Rad, Marnes-la-Coquette, France) was sequenced using MiSeq platform (Illumina, Inc., San Diego, CA, USA) paired-end sequencing and automated cluster generation with dual-indexed 2 × 250-bp reads. A 535,000/mm^2^ cluster density filter and a 96.3% cluster passing quality control filter were applied. A total of 8,690,521 reads were assembled using SPAdes version 3.12.0 with the option “–careful” in order to reduce the number of mismatches and short indels (6). Default parameters for k values, i.e., k-mer values of 127, 99, 77, 55, 33, and 21, were applied. A total of 977 human DNA contigs were removed via a BLASTn script that connected remotely to the NCBI database (nr/nt) (BLAST+ 2.3.0). Annotation using Prokka version 1.13 (7) of the *M. tilburgii* strain MEPHI genome sequence (mean coverage depth, 6.5×) comprises 102 contigs, with a total assembly size of 3,238,527 bp and 66.3% GC content (*N*_50_ value, 56,170 bp; coverage, 5.6×). One 19,595-bp contig (62.9 GC% content), exhibited 77% nucleotide similarity with Mycobacterium sp. strain KMS plasmid pMKMS02 (GenBank accession no. CP000520) using BLASTn. The *M. tilburgii* strain MEPHI genome was predicted to contain 4,207 genes, including 4,154 protein-coding genes, and 53 RNAs (49 tRNAs, 3 rRNAs, and one transfer-messenger RNA [tmRNA]). Phylogenetic trees based on the 3,504-bp *rpoB* and the 1,523-bp 16S RNA genes confirmed phylogenetic placement into the Mycobacterium simiae complex (Fig. 1B and C). The genomic similarities estimated using OrthoANI version 0.93.1 (8) and *in silico* DNA-DNA hybridization estimated using the GGDC version 2.0 online tool (9) were 87.17% and 33.8% with M. simiae (GenBank assembly accession no. GCA_000455305), 87.15% and 33.6% with Mycobacterium sherrisii (GenBank assembly accession no. GCA_001722325), 82.88% and 26.8% with Mycobacterium triplex (GenBank assembly accession no. GCA_000689255), 82.76% and 26.6% with Mycobacterium florentinum (NCBI RefSeq accession no. NZ_LQOV00000000), 82.62% and 26.1% with Mycobacterium genavense (NCBI RefSeq accession no. NZ_JAGZ00000000), 82.42% and 26.3% with Mycobacterium ahvazicum (NCBI RefSeq accession no. NZ_FXEG00000000), and 82.35% and 26% with Mycobacterium lentiflavum (NCBI RefSeq accession no. NZ_CTEE00000000), respectively. Roary pangenome analysis (10) of the M. simiae complex including *M. tilburgii* strain MEPHI yielded a total of 37,895 genes distributed as 1,077 core genome genes, 403 soft-core genes, and 5,547 shell genes (Fig. 1D). PathogenFinder 1.1 (11) predicted *M. tilburgii* strain MEPHI to be a human pathogen (probability, 0.736) with detection of 14 pathogenicity-associated proteins. Analysis using the Resistance Gene Identifier version 5.0.5 database (12) indicated that the *M. tilburgii* strain MEPHI genome harbored *rpbA*, *murA* (C117D), *mtrA*, *katG* (I335T), and *pncA* (H82R) mutations conferring resistance to rifampin, fosfomycin, macrolides, penam, isoniazid, and pyrazinamide, respectively. The whole-genome sequence confirms that *M. tilburgii* belongs to the M. simiae complex. Targeted molecular assays could be designed for the detection of *M. tilburgii* in clinical specimens.

### Data availability.

Illumina raw sequences and the assembled whole-genome sequence are available at the EMBL/GenBank under accession no. ERR3436035 and ERP116312, respectively.
